# Comparison of Sacroiliitis Grade Readings on the Same Plain Radiographs by the Same Observer at Different Periods

**DOI:** 10.7759/cureus.45817

**Published:** 2023-09-23

**Authors:** Ahmed Cihad Genc, Aysel Toçoğlu

**Affiliations:** 1 Internal Medicine, Sakarya Educational and Research Hospital, Sakarya, TUR

**Keywords:** grade, ankylosing spondylitis, intraobserver agreement, plain radiographs, sacroiliitis

## Abstract

Background: This study aimed to investigate whether there is a difference between the readings of plain sacroiliac radiographs of patients with sacroiliitis by the same observer.

Materials and methods: In the study, we included patients diagnosed with sacroiliitis through sacroiliac MRI who had undergone plain radiographs at our center between 2015 and 2022. The radiographic grading of patients was conducted by transferring their demographic and clinical information into a computerized environment so that these details would not be identifiable. The plain radiographs were numbered, and the responses were graded as grade 0, 1, 2, 3, or 4 for the right and left sacroiliac joints. The next day, using the same procedure, the same clinician re-evaluated the same plain radiographs in a different order without viewing the previous responses. This method was employed to prevent bias. The results (kappa value) were evaluated (0.00-0.20: slight agreement, 0.21-0.40: fair agreement, 0.41-0.60: moderate agreement, 0.61-0.80: substantial agreement, 0.81-1.00: perfect agreement).

Results: The study population included 478 patients and 956 sacroiliac joints from plain radiographs, both on the right and left. Following the observer's classification of the sacroiliac joints into 0, 1, 2, 3, and 4, a moderate level of agreement was found in the second evaluation of the same observer a day later with the same grades (p<0.001, kappa: 0.576). When categorized as grade 0-1 and grade 2-4, there was moderate agreement (p<0.001, kappa: 0.519), and categorization into grades 0-2 and 3-4 showed substantial agreement (p<0.001, kappa: 0.715). Analyzing the categorization into grades 0-3 and grade 4 revealed a higher kappa value, indicating substantial agreement (p<0.001, kappa: 0.766).

Conclusion: Intraobserver interpretation of radiographs may be more accurate than the interpretation of different specialists. While interpreting plain radiographs, we observed variability between adjacent grades but less variability between distant grades. However, these results need to be validated.

## Introduction

Differentiating sacroiliitis from other ankylosing spondylitis (AS) abnormalities on plain radiographs is essential for AS classification [1,2]. Axial spondyloarthritis encompasses two categories: radiographic sacroiliitis (or AS) and non-radiographic axial spondyloarthritis [3,4]. While there have been advancements in the diagnostic landscape of spondyloarthritis, imaging techniques within diagnostic protocols are still evolving and have not yet reached their full potential [5,6]. The primary impact of AS is concentrated on the spinal column and the sacroiliac joint, culminating in joint fusion along the vertebral column and constriction of spinal mobility [7,8]. Timely identification and initiation of early therapeutic strategies are imperative for individuals afflicted with these conditions, facilitating the prevention and management of concurrent ailments and averting prospective functional impairment. Diagnostic protocols, beyond the patient's clinical history, incorporate imaging methods such as sacroiliac joint radiography and sacroiliac magnetic resonance imaging (MRI). Diagnostic frameworks like the modified New York criteria still rely on radiographic benchmarks to diagnose AS [9]. Sacroiliac joint radiography entails a gradation system: grade 1 indicating no substantial abnormalities, grade 2 denoting slight localized erosion or sclerosis devoid of joint space alterations, grade 3 encompassing joint area enlargement along with narrowing or partial ankylosis coupled with erosion and sclerosis, and grade 4 indicating complete ankylosis [10,11].

There is only moderate agreement on the recognition of radiographic sacroiliitis by local readers or trained rheumatologists/radiologists. For instance, according to modified New York criteria, a significant proportion of patients with locally recognized AS were not confirmed to have AS by trained readers [12-15]. Due to the anatomical complexity of the sacroiliac joints, different doses of radiographs, and uneven articular surfaces, the possibility of misdiagnosis, especially in the early stages of sacroiliitis, has been reported, even when read by professional readers [15-17]. 

Given the diagnostic complexities of sacroiliitis, most studies, to our knowledge, have focused on evaluations by either two different radiologists or two different rheumatologists. In this study, for the first time to the best of our knowledge, we uniquely aim to analyze the consistency of readings from the same sacroiliac radiographs of patients diagnosed with sacroiliitis, taken at different times, by the same observer.

## Materials and methods

Including and excluding criteria

This study is a retrospective cross-sectional study that covers the period between 2015 and 2022. The study population consisted of 478 patients and included a total of 956 sacroiliac joints, both right and left. Medical records of patients evaluated for sacroiliitis by the Internal Medicine and Rheumatology Departments were reviewed. Patients aged 18 and older, with plain radiography, and those diagnosed with sacroiliitis through sacroiliac MRI were included in the study. Sacroiliac MRI was interpreted according to the Assessment of Spondylo Arthritis International Society (ASAS) criteria for sacroiliac joints. Patients without sacroiliac MRI or sacroiliac joint radiography or those not meeting the ASAS criteria were excluded from the study. The plain radiographs of the patients included in the study were graded according to the modified New York criteria [10,11].

Staging of sacroiliitis with plain radiograph

Sacroiliac right and left joints were staged into grades 0, 1, 2, 3, and 4 according to New York criteria. Patient identification information was covered to ensure objectivity during staging. A single reader evaluated the same image once on different days. Each plain radiograph was evaluated for up to one minute, and both sacroiliac joints were graded separately. The next day, the order of the sacroiliac joint radiographs was changed and staged again by the same observer. This method was employed to mitigate bias in assessing the interobserver agreement, which involves the consistency of plain radiograph interpretation by the same observer on two separate days. First, the analysis of the first and second intraobserver readings of the plain graphs was evaluated when the sacroiliac joints were divided into degrees 0, 1, 2, 3, and 4, and then the first and second intraobserver readings of the graphs were compared by grouping the degrees among themselves (grades 0-1 and 2-4; grades 0-2 and 3-4; grades 0-3 and 4), respectively.

Statistical analyses

Descriptive analyses were carried out to provide a comprehensive overview of the fundamental characteristics of the study population. A combination of visual techniques (such as probability plots and histograms) and analytical tests (including the Shapiro-Wilk and Kolmogorov-Smirnov tests) were utilized to assess the normal distribution of age (year). Additionally, comparisons of categorical data were executed through the utilization of the Chi-square test. The concurrence between the two interpretations was gauged using Kappa (k) coefficients. Statistical significance was acknowledged at a p-value below 0.05. These analyses were conducted using a software application (IBM SPSS Statistics, Version 22.0. Armonk, NY: IBM Corp.). The results were evaluated (0.00-0.20: slight agreement, 0.21-0.40: fair agreement, 0.41-0.60: moderate agreement, 0.61-0.80: substantial agreement, 0.81-1.00: perfect agreement).

## Results

**Figure 1:**
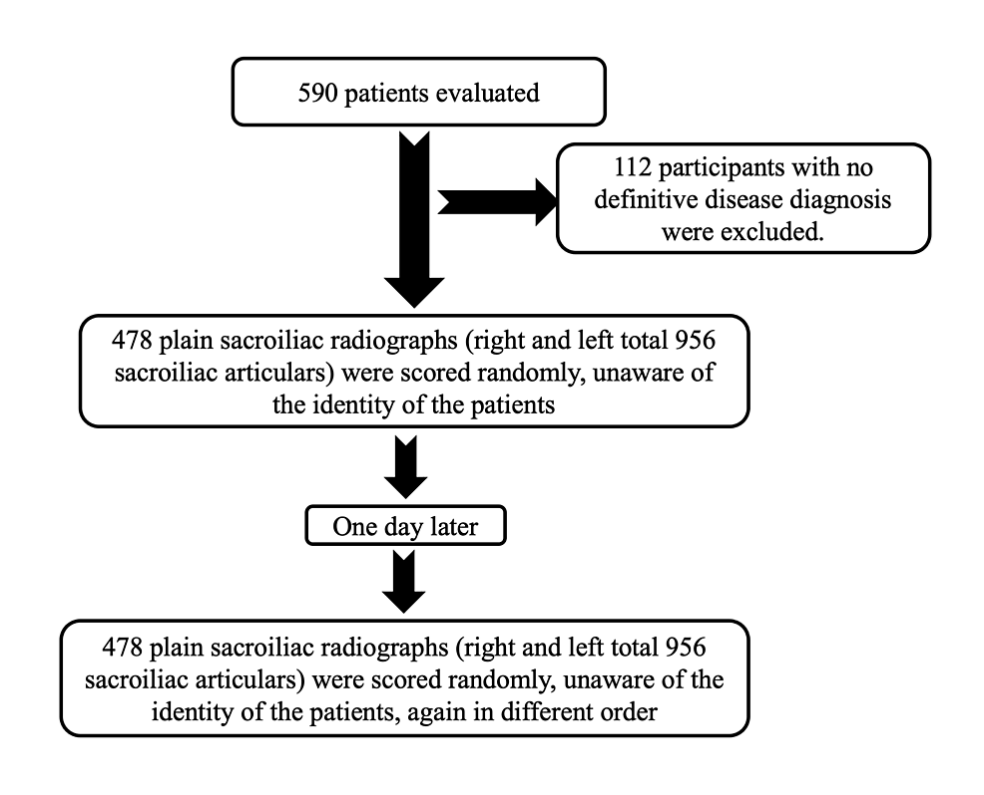
The study flowchart

The study population consisted of 478 patients, 226 (47.3%) female and 252 (52.7%) male patients. The mean age of the patients included in the study was 33±9 years. The study flow chart is shown in Figure 1.

Individual grade evaluations

First, the analysis of the first and second intraobserver readings of the plain graphs was evaluated when the sacroiliac joints were divided into degrees 0, 1, 2, 3, and 4. Table 1 presents the numbers of patients in the first and second reading for grades 0, 1, 2, 3, and 4. In the first evaluation, 33 sacroiliac joints (right or left) were evaluated as grade 0, 72 sacroiliac joints were evaluated as grade 1, 136 sacroiliac joints were graded as grade 2, 367 sacroiliac joints were graded as grade 3, and 348 sacroiliac joints were graded as grade 4. When the same observer took the same radiographs a day later, some of the evaluations were altered (Table 1). There was a statistically significant moderate agreement between the two evaluations conducted a day apart (p<0.001, k:0.576). Considering that the transition between neighboring phases was high and the characteristics of distant phases might be different, two-category analyses were performed.

**Table 1:** Analysis of intraobserver reading of flat graphs for the first and second time when sacroiliac joints are divided into grades 0, 1, 2, 3, and 4

n=478x2		First							
		Grade 0	Grade 1	Grade 2	Grade 3	Grade 4	Total	p	kappa
Second	Grade 0	8 (24.2%)	7 (9.7%)	0 (0.0%)	0 (0.0%)	0 (0.0%)	15 (1.6%)	<0.001	0.576
	Grade 1	14 (42.4%)	17 (23.6%)	12 (8.8%)	2 (0.5%)	0 (0.0%)	45 (4.7%)		
	Grade 2	7 (21.2%)	37 (51.4%)	67 (49.3%)	22 (6.0%)	0 (0.0%)	133 (13.9%)		
	Grade 3	4 (12.1%)	11 (15.3%)	57 (41.9%)	312 (85.0%)	70 (20.1%)	454 (47.5%)		
	Grade 4	0 (0.0%)	0 (0.0%)	0 (0.0%)	31 (8.4%)	278 (79.9%)	309 (32.3%)		

Two-category analysis

The results were reanalyzed as grades 0-1 and 2-4 (Table 2). In the initial evaluation, 105 sacroiliac joints were evaluated as grade 0-1. In the second evaluation, 46 (43.8%) patients were evaluated as grade 0-1, and 59 (56.2%) were evaluated as grade 2-4. In the initial evaluation, 851 sacroiliac joints were evaluated as grade 2-4. In the second evaluation, 14 (1.6%) patients were evaluated as grade 0-1 and 837 (98.4%) as grade 2-4. A statistically significant difference was also found in the analysis of stage 0-1 and stage 2-4 (p<0.001, k:0.519). When the grades 0-2 and 3-4 were compared, the agreement between the observers was calculated as substantial agreement (p<0.001, k:0.715) (Table 3).

**Table 2:** Analysis of intraobserver reading of flat graphs for the first and second time when sacroiliac joints are divided into grades 0-1 and 2-4

n=478x2		First				
		Grade 0-1	Grade 2-4	Total	p	kappa
Second	Grade 0-1	46 (43.8%)	14 (1.6%)	60 (6.3%)	<0.001	0.519
	Grade 2-4	59 (56.2%)	837 (98.4%)	896 (93.7%)		

**Table 3:** Analysis of intraobserver reading of flat graphs for the first and second time when sacroiliac joints are divided into grades 0-2 and 3-4

n=478x2		First				
		Grade 0-2	Grade 3-4	Total	p	kappa
Second	Grade 0-2	169 (70.1%)	24 (3.4%)	193 (20.2%)	<0.001	0.715
	Grade 3-4	72 (29.9%)	691 (96.6%)	763 (79.8%)		

Finally, grade 0-3 and grade 4 stages, the most distant neighbors, were statistically analyzed. In the initial evaluation, 608 sacroiliac joints were evaluated as grade 0-3. In the second evaluation, 577 (94.9%) of these patients were evaluated as grade 0-3 and 31 (5.1%) as grade 4. In the initial evaluation, 348 sacroiliac joints were evaluated as grade 4. In the second evaluation, 70 (20.1%) patients were evaluated as grade 0-3 and 278 (79.9%) as grade 4. Grouping as grade 0-3 and grade 4 showed that the intraobserver agreement approached near-perfect levels (p<0.001, k:0.766) (Table 4).

**Table 4:** Analysis of intraobserver reading of flat graphs for the first and second time when sacroiliac joints are divided into grades 0-3 and 4

n=478x2		First				
		Grade 0-3	Grade 4	Total	p	kappa
Second	Grade 0-3	577 (94.9%)	70 (20.1%)	647 (67.7%)	<0.001	0.766
	Grade 4	31 (5.1%)	278 (79.9%)	309 (32.3%)		

## Discussion

In this study, we found heterogeneity in the grading of sacroiliitis due to reading the intraobserver at different times in patients diagnosed with sacroiliitis by plain radiograph. To the best of our knowledge, the main difference between the method of this study and the methods of other studies in the literature was that the radiographs of the patients were read by the intraobserver rather than by different specialists [12,13]. Therefore, the diagnosis and grading of sacroiliitis could be more effective.

In a study, the agreement between radiograph interpretation by different readers as local and central readers was moderate (kappa: 0.54); according to local reading, 26.6% of patients had sacroiliitis, while according to central reading, 21.1% of patients had sacroiliitis. In other words, a significant proportion of patients with sacroiliitis interpreted by local readers were not confirmed to have sacroiliitis by central reading (false positive). In contrast, a small proportion had a false negative reading. These results may lead to the search for new sacroiliitis assessment criteria [12].

To determine if observer consistency was influenced by transitions between grades, we noted increased agreement when grades 0 through 3 were combined and compared with grade 4. In this case, we observed more transitivity in the near phases. However, observer agreement increased for distant grades, like between grade 1 and grade 4, moving statistically from moderate to a near-perfect agreement.

The presence of sacroiliitis is an essential criterion for the diagnosis of AS. Unilaterally or bilaterally, mild to severe inflammation of the sacroiliac joints can lead to ankylosis. Therefore, the recognition of sacroiliitis is often considered difficult and requires experience [14]. Researchers had shown significant intraobserver differences when rheumatologists and radiologists were given specialized training for better reading of radiographs, and technical training had no positive contribution to correct interpretation performance [15]. Our study did not have a unique training program for intraobservers.

The diagnosis of sacroiliitis can be made with plain radiographs, computed tomography (CT), or MRI. The sensitivity and specificity of these methods vary [18,19]. According to one study, the sensitivity and specificity were 79.1% and 70.5% for plain radiography and 82.1% and 100%, respectively, for abdominal CT [18].

In many branches of medicine, just like in most other fields, artificial intelligence-based research is rapidly growing in the fields of radiology and rheumatology as well. The utilization of artificial intelligence in radiographic interpretation can lead to enhanced intraobserver agreement, as it assists individual clinicians in maintaining consistency in their assessments over time. Additionally, it significantly improves interobserver agreement by minimizing variations in interpretations among different healthcare providers. This technological integration not only promises to enhance the overall quality of patient care but also accelerates the diagnostic process, ultimately leading to more effective and efficient healthcare practices in the field of radiology and beyond. In a study involving a total of 1553 patients, artificial intelligence has been employed to assess the sacroiliac joint. The test data detected sacroiliitis with a sensitivity of 88% and specificity of 92%. Statistical analysis of the agreement between artificial intelligence and reference judgment revealed an absolute agreement [20].

The present study had limitations, such as its retrospective nature. Although the diagnosis of sacroiliac disease is made by MRI, MRI or CT interpretations are not included in the article.

## Conclusions

In conclusion, sacroiliitis is a slowly progressing disease with a subtle clinical presentation, often leading to delayed diagnosis by a few years. Plain radiographs are the most accessible method for diagnosis. Interobserver agreement in the assessment of plain radiographs is not absolute. However, intraobserver agreement may be better compared to interobserver agreement. While interpreting plain radiographs, we observed variability between adjacent grades but less variability between distant grades. Consequently, we found a statistically significant increase in intraobserver agreement in the interpretation between grades 0-3 and grade 4, highlighting the significance of assessing these gradations.

Integrating technologies like artificial intelligence into medicine may be necessary to enhance the intraobserver and interobserver agreement in interpreting plain radiographs. Even without radiological findings, clinical follow-up should be continued for patients suspected of sacroiliitis. However, intraobserver interpretation of plain radiographs may be more accurate than interobserver or local/training reader interpretation. Randomized, prospective, and controlled studies are needed to clarify this shortcoming.
